# How common is ecological speciation in plant-feeding insects? A 'Higher' Nematinae perspective

**DOI:** 10.1186/1471-2148-10-266

**Published:** 2010-09-01

**Authors:** Tommi Nyman, Veli Vikberg, David R Smith, Jean-Luc Boevé

**Affiliations:** 1 Department of Biology, University of Eastern Finland, P.O. Box 111, FI-80101 Joensuu, Finland; 2 Liinalammintie 11 as. 6, FI-14200 Turenki, Finland; 3 Systematic Entomology Laboratory, PSI, Agricultural Research Service, US Department of Agriculture, c/o National Museum of Natural History, Smithsonian Institution, P.O. Box 37012, MRC 168, Washington, DC 20013-7012, USA; 4 Department of Entomology, Royal Belgian Institute of Natural Sciences, Rue Vautier 29, B-1000 Brussels, Belgium

## Abstract

**Background:**

Ecological speciation is a process in which a transiently resource-polymorphic species divides into two specialized sister lineages as a result of divergent selection pressures caused by the use of multiple niches or environments. Ecology-based speciation has been studied intensively in plant-feeding insects, in which both sympatric and allopatric shifts onto novel host plants could speed up diversification. However, while numerous examples of species pairs likely to have originated by resource shifts have been found, the overall importance of ecological speciation in relation to other, non-ecological speciation modes remains unknown. Here, we apply phylogenetic information on sawflies belonging to the 'Higher' Nematinae (Hymenoptera: Tenthredinidae) to infer the frequency of niche shifts in relation to speciation events.

**Results:**

Phylogenetic trees reconstructed on the basis of DNA sequence data show that the diversification of higher nematines has involved frequent shifts in larval feeding habits and in the use of plant taxa. However, the inferred number of resource shifts is considerably lower than the number of past speciation events, indicating that the majority of divergences have occurred by non-ecological allopatric speciation; based on a time-corrected analysis of sister species, we estimate that a maximum of *c*. 20% of lineage splits have been triggered by a change in resource use. In addition, we find that postspeciational changes in geographic distributions have led to broad sympatry in many species having identical host-plant ranges.

**Conclusion:**

Our analysis indicates that the importance of niche shifts for the diversification of herbivorous insects is at present implicitly and explicitly overestimated. In the case of the Higher Nematinae, employing a time correction for sister-species comparisons lowered the proportion of apparent ecology-based speciation events from *c*. 50-60% to around 20%, but such corrections are still lacking in other herbivore groups. The observed convergent but asynchronous shifting among dominant northern plant taxa in many higher-nematine clades, in combination with the broad overlaps in the geographic distributions of numerous nematine species occupying near-identical niches, indicates that host-plant shifts and herbivore community assembly are largely unconstrained by direct or indirect competition among species. More phylogeny-based studies on connections between niche diversification and speciation are needed across many insect taxa, especially in groups that exhibit few host shifts in relation to speciation.

## Background

Ecological speciation is a process in which a shift in resource or habitat use within an ancestral species triggers the formation of two new sister species, each adapted to exploit different niches [1,2]. The speciation process is thought to involve an initial period of resource polymorphism, during which the parent lineage utilizes multiple environments or niches [3,4]; subsequently, tradeoffs in the efficiency by which individuals can use different resources lead to disruptive selection and, eventually, to lineage splitting [5,6]. Ecological speciation has recently been focus of intensive research, and it is becoming increasingly clear that niche-based selection may underlie or at least speed up the diversification of, for example, many bird [7,8], lizard [9], fish [10], and invertebrate [11,12] groups.

Many of the best examples of species pairs that may have formed as a result of ecological speciation come from plant-feeding insects [2,13,14]. Insect herbivores are typically highly specialized in their host-plant preferences, and exhibit elaborate physiological [15], behavioural [16], and morphological [17] adaptations for utilizing their respective host plants. However, although only a small fraction of available plants constitute a suitable food source for most insect species, contrasts of herbivore *versus *plant phylogenies have in many cases revealed drastic discrepancies between the phylogenetic trees [18-20]. In essence, this means that host-plant associations are evolvable and change occasionally during the evolutionary history of insect lineages [21-23]. Occasional colonizations of novel hosts can theoretically cause ecological speciation, which could provide an explanation for the enormous species diversity of plant-feeding insects on the Earth [24-26].

A considerable proportion of evolutionary insect-plant research has been devoted to the possibility that ecological speciation in plant-feeding insects occurs in sympatry, so that both sister lineages are formed within a continuous geographical area [4,13,27]. However, a more likely scenario is that ecological speciation is initiated in allopatric or partially allopatric (para- or peripatric) settings; in these cases, increasing specialization onto different hosts in different parts of the geographical range of an insect species is thought to reduce the probability of hybridization if the populations later come into contact again [2,28]. Hence, the main difference to 'ordinary' allopatric speciation is that the cause for reproductive isolation lies in the disparate ecology of the incipient species, rather than in gradual accumulation of genetic incompatibilities between geographically isolated populations [29,30].

**Figure 1:**
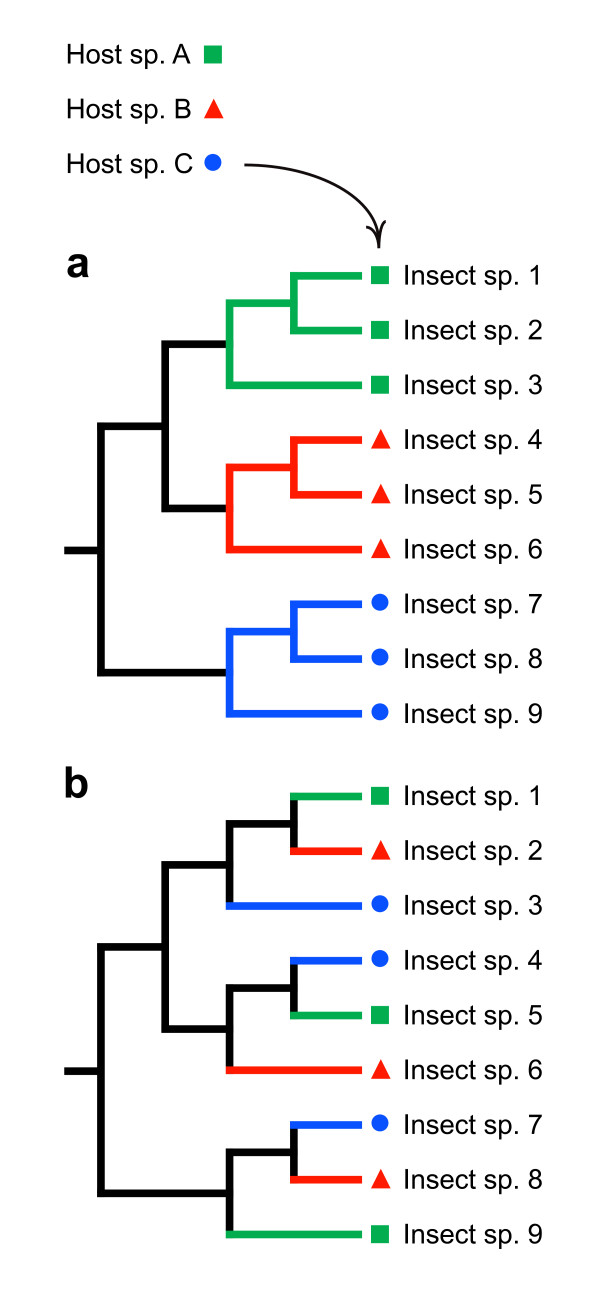
**Phylogenetic distributions of host-plant taxa arising from different speciation modes in insects**. (**a**) Distribution of host-plant taxa on the phylogeny of a hypothetical insect group in which speciation is mainly allopatric, and in which host shifts occur relatively infrequently in relation to speciation events. (**b**) Distribution of host taxa when speciation is mainly associated with host shifts. Note that only two host shifts are needed to explain current host-plant associations in **a**, whereas a minimum of six changes are needed to produce the pattern in **b**, although the number of speciation events is eight in both cases.

While numerous putative cases of ecology-based speciation in plant-feeding insects are known, we still lack an understanding of the actual frequency or importance of ecological shifts in the formation of new species [22,27,31]. Investigations of ecologically divergent species pairs can provide insights into traits or circumstances that enhance the likelihood of niche-based divergence, but studying the frequency of ecological speciation requires use of a broader, phylogeny-based approach [22,30,32,33]. The usefulness of phylogenies stems from the fact that different speciational processes should produce very different distributions of niches on the phylogenetic trees of insect herbivores: if speciation is mainly allopatric and non-ecological, particular host taxa should be closely clustered on the tips of the insect phylogeny (Fig. 1a). Conversely, if speciation is mainly ecology-based, closely related insects should tend to have different hosts, meaning that plants would occur in an intermixed fashion along the tips of the insect phylogeny (Fig. 1b). In the first case, the number of inferred host-plant shifts should be distinctly lower than the number of speciation events that are required to produce the extant herbivore species, whereas, in the latter case, the number of inferred host shifts should be close to the number of lineage splits.

**Figure 2:**
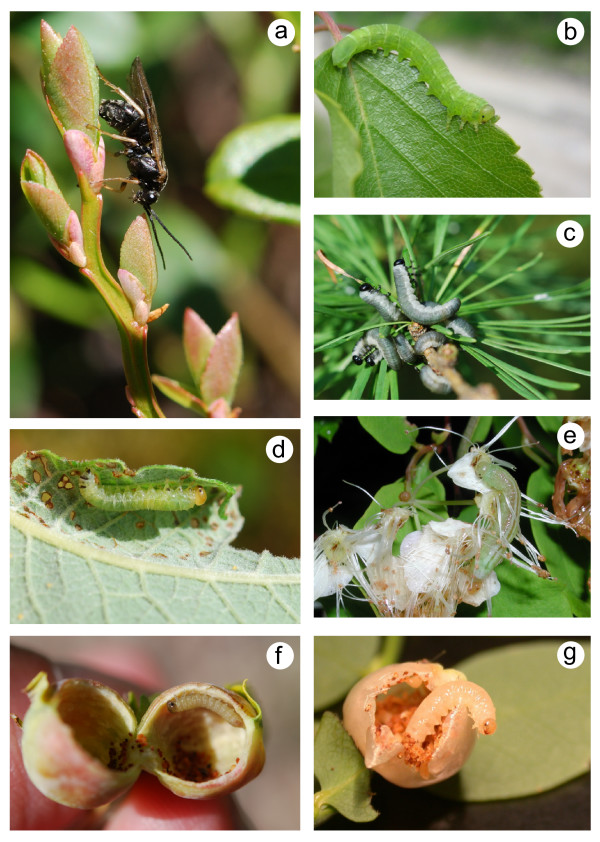
**Examples of the diversity of resource use within the Higher Nematinae**. (**a**) Female of *Pristiphora mollis *ovipositing on a leaf of *Vaccinium myrtillus*. (**b**) Larva of *Amauronematus amplus *feeding on *Betula pubescens*. (**c**) Colony of *Pristiphora erichsonii *larvae on *Larix *sp. (**d**) Larva of *Phyllocolpa leucosticta *inside opened leaf fold on *Salix caprea*. (**e**) Larva of *Pristiphora angulata *feeding on flowers of *Spiraea chamaedryfolia*. (**f**) Larva of *Pontania pustulator *inside opened leaf gall on *Salix phylicifolia*. (**g**) *Melastola *sp. larva inside opened berry of *Vaccinium parvifolium*. The locations of these exemplar species on the phylogeny of Higher Nematinae are indicated by letters in Fig. 3. (Photographs by T. Nyman).

Here, we apply a molecular phylogenetic analysis of sawflies belonging to the so-called 'Higher' Nematinae (Hymenoptera: Tenthredinidae) to estimate the relative importance of ecological *versus *non-ecological speciation in plant-feeding insects. This group of over 700 species comprises most of the taxonomic and ecological diversity found within the tenthredinid subfamily Nematinae, which prompted Ross [34] to speculate: "The higher group of genera must have evolved some highly beneficial biological characteristics, because they are at present the most abundant boreal sawfly group in number of species and probably also in population." Indeed, higher nematines are ubiquitous in most habitats across the Northern Hemisphere, and their larvae feed on a wide variety of northern plant taxa [35-37]. Their larval feeding habits are equally diverse: in addition to 'normal' external folivores, the group includes berry miners, flower and catkin feeders, leaf folders, and various gall-inducing species (Fig. 2) [38]. Combined with the high species number, such broad diversity in species-specific resource use presents many possibilities for studying the tempo and mode of speciation.

## Methods

### Taxon sampling, amplification, and sequencing

Our study builds on a previous phylogenetic analysis of the whole subfamily Nematinae [39] by adding 78 new species and sequences of a third gene (Cytochrome b) to the published dataset. The current taxon sample includes 127 exemplars of 125 Higher Nematinae species, meaning that nearly all higher-nematine species groups and main ecological niches (host-plant taxa and larval habits) are represented [35,36]. Multiple representatives were included for all large genera and species groups (Additional file 1). Trees were rooted by including three non-nematine tenthredinids and ten species belonging to the nematine tribes Hoplocampini, Stauronematini, Pseudodineurini, Caulocampini, Susanini, Dineurini, and Cladiini as outgroups in the analyses. These small 'Lower' Nematinae groups form a paraphyletic grade with respect to the ingroup [39].

Sequence data were collected from two mitochondrial genes (Cytochrome oxidase I [CoI]: 810 bp; Cytochrome b [Cytb]: 718 bp) and from two exons (501 bp + 276 bp = 777 bp) of the F2 copy of the nuclear Elongation factor-1α (EF-1α) gene following previously-described protocols [39,40]. The concatenated data matrix consists of 2305 bp of sequence data for 140 species. Sequences are missing for three, nine, and six species for CoI, Cytb, and EF-1α, respectively, but every included species has full-length sequences from at least two genes. New sequences have been submitted to GenBank under accession numbers HM237366-HM237589, and the Nexus-formatted data matrix, together with resultant phylogenetic trees, is available as Additional file 2.

### Phylogenetic analyses

**Figure 3:**
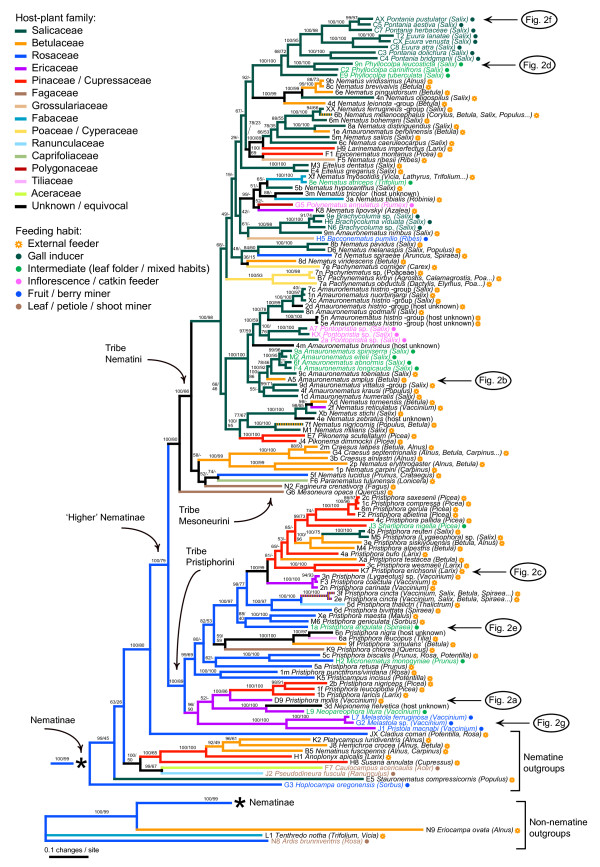
**Phylogeny of the Higher Nematinae and the diversification of host-plant use within the group**. The tree was reconstructed according to a Bayesian phylogenetic analysis allowing a separate GTR+I+Γ_4 _model of substitution for each gene. Numbers above branches show Bayesian posterior probabilities (%) followed by bootstrap proportions (%) from the corresponding ML analysis (hyphens in the place of bootstrap values denote clades that were not present in the ML tree). Branches are colored according to a maximum-parsimony reconstruction of host-family use, larval feeding habits are indicated by font colors and by symbols after species names (see legend). Species illustrated in Fig. 2 are indicated to the right of the tree.

Modeltest 3.5 [41] was implemented in conjunction with PAUP* 4.0b10 [42] to identify the least complex substitution model for use in Bayesian phylogenetic analyses in MrBayes 3.1.2 [43]. Hierarchical likelihood ratio tests indicated a GTR+I+Γ_4 _model as optimal for each of the three genes. A separate, unlinked substitution model was allowed for each gene in a three-partition analysis. A single run employing default priors was run for eight million generations with eight incrementally heated (t = 0.1) chains; tree sampling was done from the current cold chain every 100th generation, and the first 10,001 trees recovered prior to reaching stationarity were discarded as a burnin. The consensus tree showing all compatible groupings (Fig. 3) was calculated on the basis of the remaining 70,000 trees. A corresponding maximum-likelihood (ML) analysis was performed using RAxML 7.0.4 [44]. This analysis employed a separate GTR+I+Γ_4 _model for each gene, but branch lengths were estimated jointly for the whole data (Additional file 2). Clade support was estimated on the basis of 500 bootstrap replicates of the data matrix (Fig. 3).

**Figure 4:**
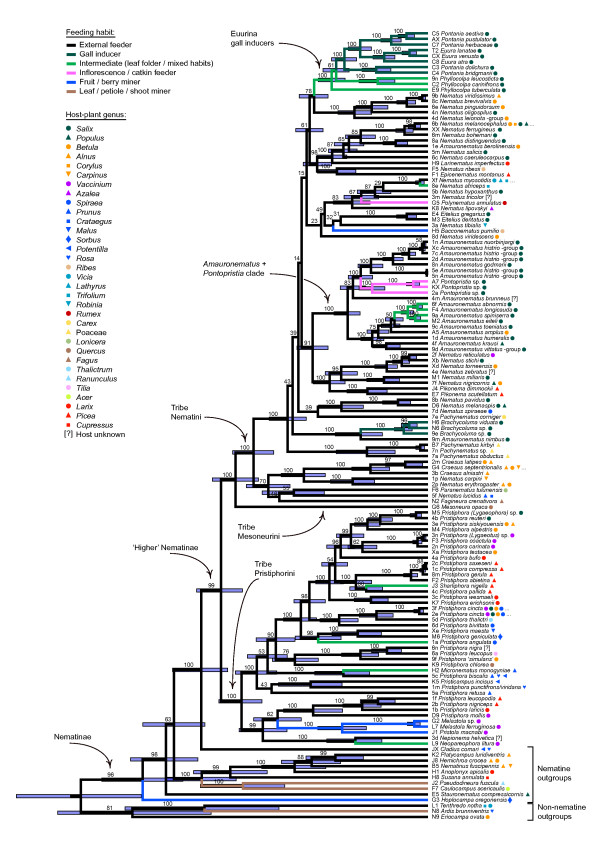
**Relaxed molecular-clock phylogeny of the Higher Nematinae, and the evolution of different larval habits within the group**. The maximum clade credibility tree resulted from a topologically unconstrained Bayesian phylogenetic analysis employing a relaxed lognormal clock and a separate GTR+I+Γ_4 _model of substitution for each gene. Numbers above branches show posterior probabilities (%), and blue shaded bars the 95% highest posterior density intervals for relative node ages for nodes with probabilities over 50%. Branch colors denote larval feeding habits according to unordered maximum-parsimony optimization, symbols to the right of species names show host-plant genera and families of the exemplar species (see legend). Full host ranges of polyphagous species are given in Additional file 1.

BEAST 1.4.8 [45] was used to estimate the relative ages of various nematine groups based on a Bayesian relaxed molecular clock method. The topologically unconstrained analysis allowed a separate GTR+I+ Γ_4 _model of substitution for each gene and employed an uncorrelated relaxed lognormal clock model for rate variation among branches, a Yule prior on speciation, and default priors for other parameters except for the mean of branch rates (ucld.mean), which was fixed to 1. Three independent runs with automatic tuning of operators were run for 80 million generations, and parameters and trees were sampled every 1,000 generations (the XML file is available as Additional file 3). After inspection of adequate convergence of runs and effective sample sizes of the parameters in Tracer 1.4.1 [46], the tree files were combined in LogCombiner 1.4.8 (part of the BEAST package). The first 40,000 trees from each file were discarded as a burnin, and the tree file was subsequently thinned by resampling trees every 3,000 generations; the maximum clade credibility (MCC) tree showing mean branch lengths (Fig. 4) is based on the 40,001 post-stationarity trees that remained after thinning.

### Character analyses

To reconstruct ancestral host-plant families and feeding habits, these traits were treated as unordered multistate characters and maximum-parsimony optimized on the phylogenetic trees using Mesquite 2.6 [47]. Oligo- and polyphagous taxa were coded with all used host families.

To estimate the number of ecological shifts that have occurred during the radiation of the Higher Nematinae, we first identified all distinct ecological niches (feeding habit × host plant(s)) found in the ingroup species included in the phylogenetic analysis, and coded each niche with a separate state within a single character (outgroup states were coded as unknown). Because the aim was to calculate numbers of changes, the typical number of steps between two different states was 1. However, we also created 'generalist' states for species that utilize multiple plant taxa and then used the step-matrix option in Mesquite to define the cost to these states, from the plant taxa that are included within the generalist host range, as being zero. By doing so, we essentially assumed that a clear overlap in the host ranges of different species implies that they have not speciated ecologically (theoretical models of resource-based speciation typically assume distinct, non-overlapping niches as the cause of divergent selection [2,30]). Phylogenetic uncertainty in the estimate was taken into account by recording the numbers of steps in the niche character across the 70,000 post-burnin trees that were sampled by MrBayes during the phylogenetic analysis [48].

As a separate estimate of the proportion of lineage splits accompanied by a shift in resource use, we indentified all terminal sister-species pairs across the MCC tree (Fig. 4), and then separated these 35 pairs into those in which both species have identical or overlapping niches, and into those in which the species have different niches. Thereafter, we performed a logistic regression in SPSS for Windows 17.0 (SPSS, Inc., 233 S. Wacker Drive, Chicago, IL 60606-6307, USA) to test whether the probability that sister species have a different niche depends on the time elapsed since their most recent common ancestor (= relative node height in the MCC tree).

**Figure 5:**
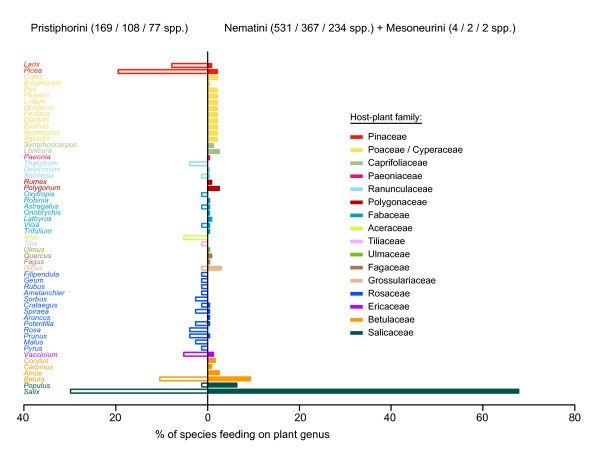
**Distributions of Higher Nematinae species on different plant genera**. Proportions are shown separately for the tribe Pristiphorini and for its sister clade composed of the tribes Mesoneurini and Nematini (see Figs. 3 and 4). Host data and estimates of species numbers are from Lacourt's [36] checklist of Western Palearctic sawflies, plant families are denoted by separate font colors (see legend). Numbers in parentheses after tribe names are in the order: total number of species/number of Western Palearctic species/number of Western Palearctic species with known hosts.

Proportions of higher nematine species feeding on different plant genera (Fig. 5) were extracted from Lacourt's [36] list of host-plant affiliations of sawflies of the Western Palearctic region. Only species with known hosts were included, and proportions were calculated separately for the tribe Pristiphorini and for the Nematini+Mesoneurini clade (see Figs. 3, 4 and 5). Oligo- and polyphagous species were counted as an additional species for each plant genus on which they feed (for example, the oligophagous *Craesus latipes *(Villaret) was treated as one species on *Alnus *and another on *Betula*).

## Results

### Phylogenetic trees

The Bayesian and ML analyses of the sequence data produced relatively well-supported trees showing that the Higher Nematinae constitutes a monophyletic clade within the subfamily Nematinae (Figs. 3 and 4; the ML tree is included in Additional file 2). The three topologies are largely congruent, with discrepancies mainly evident in tree regions that are weakly supported. Major divisions within the ingroup correspond closely with the traditional tribes Nematini, Pristiphorini, and Mesoneurini (Figs. 3 and 4). Conflict with traditional classifications is mostly evident in that the largest nematine genera (*Nematus*, *Pristiphora*, and *Amauronematus*) come out as para- and polyphyletic (see [39]). Posterior probabilities and bootstrap proportions of many groupings within Nematini are low, but because these uncertainties concern mainly relationships among strongly supported middle-level clades, they have only minor importance for the conclusions below.

The trees document a pattern of frequent faunal exchange across the Holarctic region, because European, North American, and Asian exemplar species (see Additional file 1) are intermixed throughout the trees. Nearctic species are found scattered among European ones in all major higher-nematine genera (*Nematus*, *Amauronematus*, and *Pristiphora*), but also in smaller groups such as *Eitelius*, *Pontopristia*, and *Pikonema *(Figs. 3 and 4). Our analysis also confirms Smith's [49] hypothesis of a close relationship between the North American *Nematus erythrogaster *-group and the Holarctic genus *Craesus*, as well as his earlier [50] suggestion of a connection between the exclusively Nearctic genus *Neopareophora *and European *Nepionema*.

### Evolutionary dynamics of resource use

Diversification of the Higher Nematinae has been a dynamic process in which host-plant associations and larval lifestyles change continually, although feeding-habit changes are distinctly rarer than shifts in host-plant use (Figs. 3 and 4). Various forms of internal feeding, such as gall induction, leaf folding, catkin feeding, and berry mining, have evolved repeatedly from ancestors whose larvae were external feeders on leaves or on needles (Fig. 4). Higher-nematine larvae are currently found on plants belonging to over 16 families, but most species are concentrated on plants in Salicaceae, Betulaceae, Rosaceae, Ericaceae, and Pinaceae (Fig. 5), partly because colonization and recolonization events have occurred repeatedly among these plant taxa (Fig. 3). Interestingly, the distribution of species across utilized plant families differs between the clade formed by Pristiphorini and its sister clade formed by Nematini+Mesoneurini (Fig. 5; *χ*^2^= 155.93, df = 15, *P *< 0.0001), the clearest discrepancy being the high proportion of *Salix*-feeding species within the most species-rich tribe Nematini.

### Speciation and niche shifts

The existence of the 125 sampled ingroup species demands 124 past speciation events, but explaining the current distribution of species-level niches on the Bayesian consensus tree (Fig. 3) and on the ML tree requires only 68 shifts in feeding habits and/or host taxa. This estimate is robust against phylogenetic uncertainty, because when niches are similarly maximum-parsimony optimized on the 70,000 Bayesian post-burnin trees, 67-69 shifts (mean = 68.13) are needed. Even if shifts to generalist host-use states are treated as a true niche shift (= 1 step from other states), the Bayesian consensus tree is only 75 steps long (ML tree = 74 steps), and all trees in the Bayesian tree sample require between 73 and 75 changes (mean = 74.73 steps).

**Figure 6:**
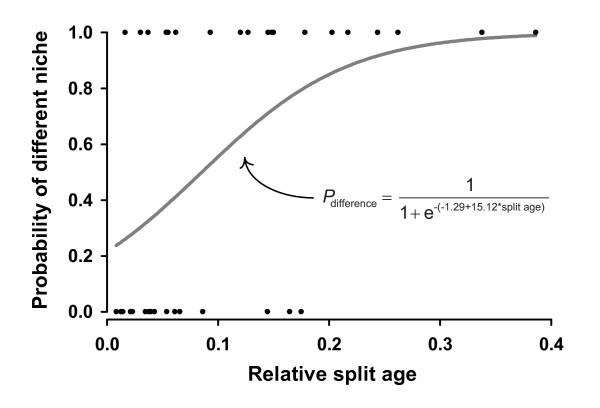
**The probability that higher-nematine sister species have different niches in relation to time since their divergence**. Data on pairwise niche differences (1 = different hosts and/or larval feeding habits; 0 = identical or overlapping niches) and split ages (= relative time since common ancestor) was taken from the 35 terminal sister-taxon pairs in the Bayesian MCC tree (Fig. 4), and the probability curve was estimated using logistic regression.

When only sister-taxon pairs on the MCC tree are considered, species in 19 out of 35 pairs (54.3%) have non-overlapping host ranges and/or a distinct difference in their larval feeding modes. However, the logistic regression (Fig. 6) shows that the probability of sister species having different niches is strongly affected by the time since their most recent common ancestor: *P*_difference _= 1/(1+e^-(-1.29+15.12*split age)^), the constant (*P *= 0.045) and the effect of split age (*P *= 0.013) being statistically significant.

## Discussion

Research on ecological speciation has traditionally focussed on sister-species pairs or on small groups of closely related lineages that differ in their resource use, and which could therefore have originated by niche shifts. While such studies have convincingly shown that ecology-based diversification is possible in highly disparate taxa and under many plausible scenarios (*e.g*., [8,51,52]), the overall frequency of ecological speciation remains unknown. Recently, broader phylogenetic approaches have provided insights into the relative importance of alternative speciation modes [53-55]. For example, comparative analyses employing age-range corrections have shown that closely related species tend to have less overlap in their geographical ranges than do distantly related species, which indicates that speciation rarely occurs in sympatric settings [53,56-58]. However, the finding that speciation is largely allopatric does not exclude the possibility that the build-up of reproductive isolation between incipient species has an ecological basis: as mentioned above, ecological divergence can, and in fact is more likely to, occur in complete or partial allopatry [27,28,30]. Therefore, phylogenetic studies on the frequency of niche shifts in relation to the number of past speciation events are more likely to produce a correct view of the prevalence of ecological speciation [22,31,33,56].

The traditional paradigm in plant-herbivore research is that host shifts are a major factor promoting species divergence [23,59,60]. However, there are two good reasons for suspecting that the importance of niche shifts for speciation is overestimated: First, there has been a huge--most likely disproportionate--interest in the intriguing possibility that host-associated speciation in insect herbivores could occur in sympatry, *i.e*., without geographical isolation [4,13,27,58]. Second, it seems probable--and perfectly logical--that insect groups that are chosen for phylogenetic studies on host-plant shifts are selected preferentially from taxa in which species are known *a priori *to be relatively specialized and to exhibit clear interspecific variation in host-plant use (*e.g*., [19,40,61]).

In the case of the Higher Nematinae, our phylogenetic analysis reveals a pattern of frequent niche shifts both in terms of larval lifestyles and host-plant use (Figs. 3 and 4). Despite this, optimizing larval niches on the Bayesian tree sample shows that at most about 60% of lineage splits could have been caused by ecological factors. This value should be considered as an upper limit, because our ecologically overdispersed taxon-sampling scheme will raise the relative number of niche shifts, and the sampling design should also override the tendency of maximum parsimony to underestimate the frequency of changes in fast-evolving traits [62]. When only sister species are considered, the percentage of pairs having divergent niches is 54. This raw value is intriguingly close to Winkler & Mitter's [22] recent 'fifty-fifty' estimate of the proportions of ecological vs. nonecological speciation, which was based on a broad literature survey of sister species of herbivorous insects. However, our logistic regression (Fig. 6) shows that immediately after speciation only an estimated 21.6% of higher-nematine sister-species pairs would have non-overlapping niches. The discrepancy between the methods is most likely explained by postspeciational host shifts, which can inflate the apparent frequency of ecological speciation in uncorrected sister-species comparisons. Although denser taxon sampling will be needed for a more exact estimate of the prevalence of ecology-based diversification within the Higher Nematinae, the marked drop in the inferred proportion in the age-adjusted analysis suggests that phylogenetic time corrections would be useful also in surveys of other insect herbivore taxa.

It is possible, however, that the frequency of ecological speciation varies among clades [22,31]. In particular, it appears that the extreme diversity (400-500 species [63,64]) of gall-inducing nematines in the subtribe Euurina (Figs. 3 and 4) has been spurred by host-shifting among *Salix *species. Like other gall-inducing insects [65], Euurina gallers are very host-specific compared to willow-associated nematines having external-feeding larvae, which tend to utilize multiple host species [36,66], and which therefore probably may have radiated mainly allopatrically. Higher-nematine subgenera and species-groups feeding externally on other plant taxa are likewise often dominated by species having identical or broadly overlapping host-plant ranges [36,38,66], and niche shifting seems to be particularly infrequent in relation to speciation in groups associated with *Picea*, *Larix, Vaccinium*, and plants within Betulaceae (Fig. 3). It remains to be studied whether such non-ecological radiations can be used to estimate the proportion of non-ecological speciation in related groups in which species differ in their host use, because some proportion of host switches also in these groups undoubtedly have occurred well before or after speciation events (*cf*. [33]).

While earlier hypotheses on insect diversification emphasized coevolution of plant defenses and herbivore counterdefenses as a major driver of insect diversification [59,67,68], recent studies applying dated phylogenies have uncovered a possible role of long-term climatic conditions in determining rates of speciation and extinction in various herbivore groups [69-71]. Throughout the Earth's history, climatic changes have lead to major shifts in plant communities and global vegetation patterns, with direct negative or positive consequences for associated herbivores [72]. In particular, systems experiencing repeated cycles of range contractions, expansions, and faunal mixing can constitute 'speciation machines' that lead to escalating diversification across multiple trophic levels [73-75]. A Cenozoic high-latitude speciation machine could explain why higher nematines seem to have an inordinate fondness for willows: nearly a third of Western Palearctic species in the tribe Pristiphorini feed on *Salix *species, and in the Nematini the percentage is as high as 68 (Fig. 5; this general pattern holds also in North America [35,37]). The largest willow-associated radiations have occurred within the aforementioned gall-inducing subtribe Euurina, and in the predominantly willow-feeding *Amauronematus+Pontopristia *clade, which includes at least 112 species [36,76]. These temporally overlapping radiations began *c*. 30 million years ago (Mya), assuming that the most recent common ancestor of the Higher Nematinae lived about 70 Mya (Fig. 4; see [39]). Interestingly, this would place the onset of these radiations at the time of strong cooling of the global climate, which began in the early Oligocene *c*. 35 Mya, and which was followed by alternating periods of cold ice ages and warmer interglacials [77]. Such climatic oscillations, with resultant long-distance migrations of whole ecosystems, could have promoted the diversification of willows that are concentrated in relatively cool habitats and that currently comprise over 400 species [78,79]. The conditions that generated diversity in *Salix *would simultaneously have acted also on the insect groups that depend on them and, like in higher nematines, willows currently support a considerable proportion of species also in, for example, northern butterflies and moths [80], phytophagous beetles [80,81], and leafhoppers [31].

The role of competition in directing the historical assembly and present structure of herbivore communities has been debated for decades [82-84]. If competition was a force directing host switching, shifts would tend to occur towards un- or underused plant taxa, meaning that, over time, herbivore host-plant associations would become overdispersed with regard to plant phylogeny. By contrast, higher-nematine host use is strongly underdispersed, shifts having occurred repeatedly and in many directions among a handful of dominant northern plant families, while a large proportion of the Holarctic flora apparently has been effectively ignored for tens of millions of years. This shifting pattern conforms to the 'resource island model' [23,85,86] of herbivore diversification, in which phylogenetically biased colonizations and back-colonizations among plant taxa, in combination with abundance-dependent extinction, lead to accumulation of herbivore species on common plants that have many relatives [80,87,88]. Competition could still operate more subtly, if recruitment follows a 'macroevolutionary ideal free distribution' (*cf*. [89]), so that the number of herbivore species that can be supported depends on the commonness (or overall biomass) of a given plant [90]. However, the convergent, asynchronous, and undoubtedly ongoing colonizations of many plant taxa by various higher-nematine groups (Figs. 3 and 4) indicates that ecological pre-emption of host taxa does not occur, and that northern insect-plant communities are still unsaturated and could therefore soak up even more herbivore species in the future. The broad overlaps in the geographical distributions [35,36] of many closely related, ecologically near-identical higher-nematine species--that necessarily must have diverged in allopatry and then brought to sympatry by postspeciational range shifts--provides further support for the view that interspecific competition, either via direct resource competition or via indirect competition caused by shared natural enemies, is of minor importance in structuring herbivore communities [73,82,84,91].

## Conclusions

Our phylogeny-based analysis of the Higher Nematinae strongly indicates that the importance of niche shifts for speciation in plant-feeding insects is at present explicitly and implicitly overestimated. In particular, applying a time correction for sister-group comparisons lowered the proportion of apparent ecology-based speciation events from roughly 50% to around 20%. The vast majority of lineage splits in higher nematines therefore seem to have occurred non-ecologically in allopatry, and this may well be true also for most other plant-feeding insects. Reconciling this result with the finding of Janz *et al. *[92] that species richness in nymphalid butterfly clades correlates positively with collective host ranges requires further work; we propose that the correlation follows from reduced extinction probabilities in ecologically versatile groups, rather than from increased ecological speciation within them.

Evolutionary dynamics observed within the Higher Nematinae favour a largely non-interactive, non-equilibrium view of community assembly in northern plant-herbivore networks: geographical shifts across the whole Northern Hemisphere have been commonplace in many higher-nematine groups, and the frequent co-occurrence of related species utilizing seemingly identical niches indicates that distributional changes occur largely unimpeded by direct or indirect competitive interactions. More detailed surveys of local communities are, however, necessary in order to exclude the possibility that competitive repulsion occurs on smaller geographical scales, which could lead to a mosaic pattern of patch occupancy by ecologically equivalent relatives [93].

The Higher Nematinae comprises well over 70% of the species in the subfamily Nematinae, but the main part of higher-nematine diversity lies within the tribe Nematini, in which a strikingly high proportion of species use willows as hosts. This suggests that the success of higher nematines was caused, not by the evolution of superior biological characteristics as suggested by Ross [34], but by a fortuitous association with willows at a time of a cyclically cooling global climate. Reliably dated molecular-phylogenetic analyses of *Salix *and *Salix*-associated herbivores are desperately needed to test our hypothesis that the diversification of willow-based food webs was accelerated during the latter half of the Cenozoic Era. The genus *Salix *has thus far proven to be an extremely challenging target for such studies [94,95], but even comparative analyses across herbivore taxa would surely provide interesting insights into the evolutionary history of Holarctic plant-herbivore communities.

## Authors' contributions

The study was conceived and designed by TN and JLB. TN performed laboratory and data analyses, prepared figures, and wrote the manuscript. JLB planned taxon sampling and study setup, and assisted in writing. VV and DRS provided taxonomic and ecological background information and identified specimens used in the analyses. All authors read and approved the manuscript.

## Supplementary Material

Additional file 1 **Collection data for exemplar specimens, and taxonomic and ecological background information**. Excel file containing collection data for the specimens used in the study, as well as species numbers, geographical distributions, larval lifestyles, and collective host ranges of genera, subgenera, and species groups within the Higher Nematinae. Click here for file

Additional file 2 **Sequence data used in phylogeny reconstruction and resultant phylogenetic trees**. NEXUS file containing the data matrix and trees obtained from the Bayesian phylogenetic analyses in MrBayes and BEAST, and the maximum-likelihood tree from the analysis using RAxML. Click here for file

Additional file 3 **Data file and run parameters for BEAST**. XML file used for the phylogenetic analysis in BEAST. Click here for file
